# Experimental and Numerical Study on Withdrawal Strength of Different Types of Auxetic Dowels for Furniture Joints

**DOI:** 10.3390/ma13194252

**Published:** 2020-09-24

**Authors:** Ali Kasal, Tolga Kuşkun, Jerzy Smardzewski

**Affiliations:** 1 Department of Woodworking Industrial Engineering, Faculty of Technology, Muğla Sitki Koçman University, Mugla 48000, Turkey; tolgakuskun@mu.edu.tr; 2 Department of Furniture Design, Faculty of Wood Technology, Poznan University of Life Sciences, Wojska Polskiego 28, 60-637 Poznan, Poland; jsmardzewski@up.poznan.pl

**Keywords:** auxetic, dowel joints, withdrawal strength, friction, contact pressure, FEM

## Abstract

Studies on the application of auxetic metamaterials and structures in furniture joints are very limited. However, they have huge potential for use in ready-to-assemble furniture. This study aimed to design and produce different types of auxetic dowels in 3D printing technology, and experimentally and numerically analyze the withdrawal strength of these dowels. In the scope of the study, 24 auxetic dowels with different types and size of inclusions, different diameter of holes, and a non-auxetic reference dowel were designed and produced with appropriate muffs. Dowels were 3D printed from polyamide (PA12). Poisson’s ratios, withdrawal strength, contact pressures, and friction coefficients of dowels were determined theoretically by means of numerical analyses and real static compression tests. After the pre-production of dowels, the dowels with triangular inclusions have not been found to have sufficient strength and stiffness. Withdrawal strength of dowels decreased as the size of inclusions is decreased, or dowel hole diameter is increased. Furthermore, contact pressures and stresses in auxetic dowels were considerably lower than non-auxetic dowels under the withdrawal force.

## 1. Introduction

The strength and stability of any structural type of furniture depends on the connections that hold its components together. Furniture components could be connected to each other with an extensive variety of glued or unglued joint techniques by using various types of fasteners, such as dowels, nails, screws, staples, eccentric connectors, metal or plastic connectors, etc. Glued dowel joints are the most popular method for joining the member together in furniture constructions. Especially, wooden dowels are commonly used to join wood and wood-based materials together in many types of furniture construction. In typical furniture, dowel joints may be subjected to axial (withdrawal), shear, torsional, and bending forces; thus, they should have sufficient strength to carry these loads.

Many studies have been done for withdrawal strength and bending moment capacities of dowels from wood and wood-based materials. Eckelman and Cassens [1] investigated the withdrawal strength of different surfaces of dowels from wood-based panels. Results indicated that the plain and spiral-groove dowels give better face withdrawal strength than multi-groove dowels, and both face and edge withdrawal strength of the dowels linearly increased as the dowel penetration increased. Furthermore, predictive expressions for edge and face withdrawal strengths were given in the study [1]. The study related to the bending moment and moment rotation characteristics of *T*-type, two-pin dowel joints indicated that the ultimate bending moment capacity (*M*) of the joint could be estimated by means of the expression *M = F* × *d*, where *F* = the ultimate direct withdrawal strength of a single dowel and *d* = the distance between resultant compression and tension forces vectors [2].

Bending moment capacity and moment-rotation characteristics of *T*-type two-pin dowel joints constructed of solid woods and wood composites were also investigated in another study. According to the test results, joints constructed of red oak and plywood had the highest bending moment resistance, and the joints of particleboard had the weakest bending resistance. Bending moment capacity of the joint could be estimated by means of the expression *M = (d*1/2 *+ w*/3 *+ e*/3*) × T*, where *T* = the ultimate direct withdrawal strength of a single dowel, *w* = the width of the rail, *e* = the distance from the rail centerline to the neutral axis and *d*1 is the spacing between two dowels [3]. A study was conducted to obtain basic information about the withdrawal strength of dowels in both plywood and oriented strand board. Results of the tests were incorporated into predictive expressions that allow designers to estimate withdrawal strength as a function of the diameter of the dowels, their depth of embedment and the density of the composite material [4]. Kasal [5] searched the edge and face withdrawal strength of dowels from some wood and wood composite materials in the other study. Ors and Efe [6] investigated the mechanical properties of furniture fasteners used in the frame construction furniture, and demonstrated that the joints constructed with minifix (threaded insert type) and multifix (knock-down type) fasteners perform better than the traditional joint (glued) types.

Glued joints have been replaced by joints with mechanical fasteners (ready to assemble joints) in the furniture constructions today. Nowadays, the development of mass production technologies and the transportation of manufactured furniture to foreign countries have made it necessary for furniture to be ready to assemble. Joints without adhesives are popular in furniture construction since their use allows the furniture to be shipped in disassembled and assembled on site, which greatly reduces shipping costs. This is an important consideration both in case of domestic and export furniture.

Conventional materials are generally used in the production of mechanical fasteners for furniture joints. However, the interest and need for smart materials is increasing day by day, which encourages the design, production, and use of new alternative materials. Smart material designs and productions are made either by developing a new product or by adding extra properties to the traditional materials. Smart materials are structures that have unusual material properties like negative Poisson’s ratio, negative thermal expansion coefficient, high bulk modulus, high shear modulus, or negative compressibility. The most popular class of the smart materials in the literature is ‘auxetic’ materials which have a negative Poisson’s ratio.

Poisson’s ratio can be described as a dimensionless constant value that depends on the direction of an applied load, and indicates the ratio of negative transverse strain to the longitudinal strain of a body subjected to a tensile load [7]. This elastic constant value provides a universal way to compare the structural performance of real homogeneous and non-homogeneous materials [8]. Poisson’s ratio was commonly expected to be positive; however, there are materials that have a negative Poisson’s ratio, and they showed different behavior. These materials expand their transverse dimension when subjected to axial tensile loads and shrink it when they compressed [9]. The atypical elastic behavior of auxetic materials is enabling advancements in a broad range of technologies such as impact-resistant composites, extremely precise sensors, tougher ceramics, and high-performance armor [10,11]. The interest in auxetic materials has been increasing in recent years; consequently, studies based on the experimental and modeling for these materials are becoming widespread. These materials could be alternative to traditional materials. The fact that the Poisson’s ratio, which has an effect on many basic properties of the materials, is negative in auxetic materials contrary to traditional materials encourages research into the use of these materials in engineering applications.

Structures with a negative Poisson’s ratio were described as early as 30 years ago [12,13,14]. Early stage studies were performed in 1987 by Lakes [9], and name “auxetics” was introduced by Evans [15]. Auxetics can be single molecules, crystals, or a particular structure of macroscopic matter. Auxetic materials and structures are expected to have many desirable mechanical properties such as shear resistance [7,16], indentation resistance [17,18], synclastic behavior [10,19], high energy absorption [20,21], and fracture resistance [9].

There are many studies on auxetic materials. In a study that was carried by Carneiro et al. [7], the theories explaining the deformation behavior of auxetic materials and revealing the important role represented by the internal structure were presented. Their mechanical properties were explored, and some potential applications of these materials were shown. Numerical analysis of mechanical behavior of auxetic structures with re-entrant cells subjected to uni-axial quasi-static compression was investigated. The mechanical behavior was evaluated inversely with respect to selected geometrical parameters of the unit cell and two different loading modes. Finite element method was used for the numerical analysis of the problem, and from results of simulations, Young’s modulus, the characteristics of Poisson’s ratio function, and deformation energy density were estimated [22]. Patiballa and Krishnan [23] presented a new mechanics-based framework for the qualitative analysis and conceptual design of mechanical metamaterials, and specifically materials exhibiting auxetic behavior. In their paper, auxetic materials were qualitatively classified into two classes, namely, high shear and low shear microstructures. Khare et al. [24] demonstrated that 3D printed structures with stress delocalization that enables macroscopic 30% cyclable elastic strains, far exceeding those intrinsic to the materials that constitute them (6%). They also presented a simple semi-analytical model of the deformations, which enables prediction of mechanical properties of the structures within <5% error of experimental measurements from a few parameters such as dimensions and material properties. Santulli and Langella [25] discussed the experience of using auxetic materials in different design objects, including chairs, bags, seat belts, etc. Structures were calculated and modeled as chiral with defined geometrical parameters and then applied to concepts with the fabrication of real models using neoprene or common rubbery material.

A limited number of papers describe the practical use of auxetics in wood and furniture industry. In the studies conducted by the Smardzewski et al. [26,27], the aim was to develop a model of an auxetic compression spring useful in seating furniture constructions. Ren et al. [28] designed, manufactured, and investigated the first auxetic nails for wood and furniture industry. The push-in and pull-out performance of auxetic and non-auxetic nails were compared by using two key parameters: namely, maximum compressive force and maximum tensile force. According to results, they found that the auxetic nails do not always exhibit superior mechanical performance to non-auxetic ones. Also, the small auxetic deformation of one typical designed auxetic nail is revealed by the experimentally validated FEM model. Finally, some suggestions were provided for more effective designs of future auxetic nails.

As seen in the literature, studies on the application of the auxetic materials in furniture joints are very limited. In this study, it was considered that the property of negative Poisson’s ratio could be used to design different kind of auxetic dowels for easier push-in and harder pull-out for the furniture joints. The designed and produced dowels could be utilized for one-time ready to assemble (RTA) furniture joints. Furthermore, 3D printing techniques may prove useful in such prototype studies. Application of the selective laser sintering (SLS) technology makes it possible to produce even highly complex prototypes of joints of a high degree of isotropy in the used material. To summarize, the aim of this study was to design and produce different kinds of auxetic dowels in 3D printing technology, then, experimentally and numerically analyze the withdrawal strength, contact pressures, and friction coefficients of these dowels in particleboard.

## 2. Materials and Methods

### 2.1. Determination of Mechanical and Elastic Properties of Dowel Material

**Figure 1:**
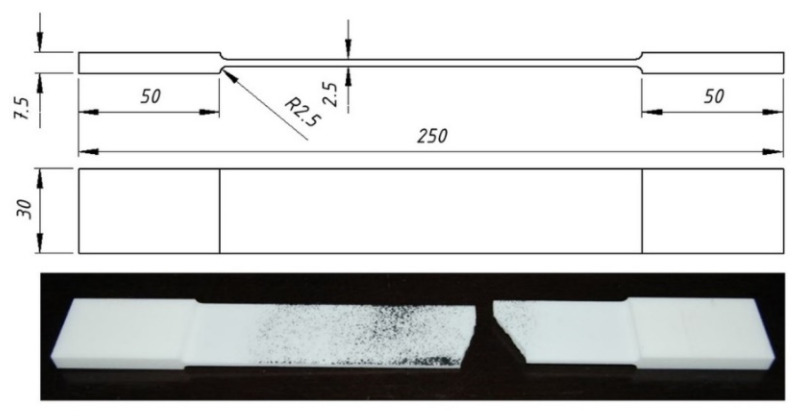
Dimensions and real picture of the tensile test samples.

The designed dowels were printed by using polyamide (PA12) (3D Center, Wrocław, Poland). The mechanical and elastic properties of the PA12 were determined according to the uniaxial tensile tests with 10 samples in accordance with the procedures described in ASTM D3039/D3039M–17 [29]. The test sample is shown in Figure 1.

**Figure 2:**
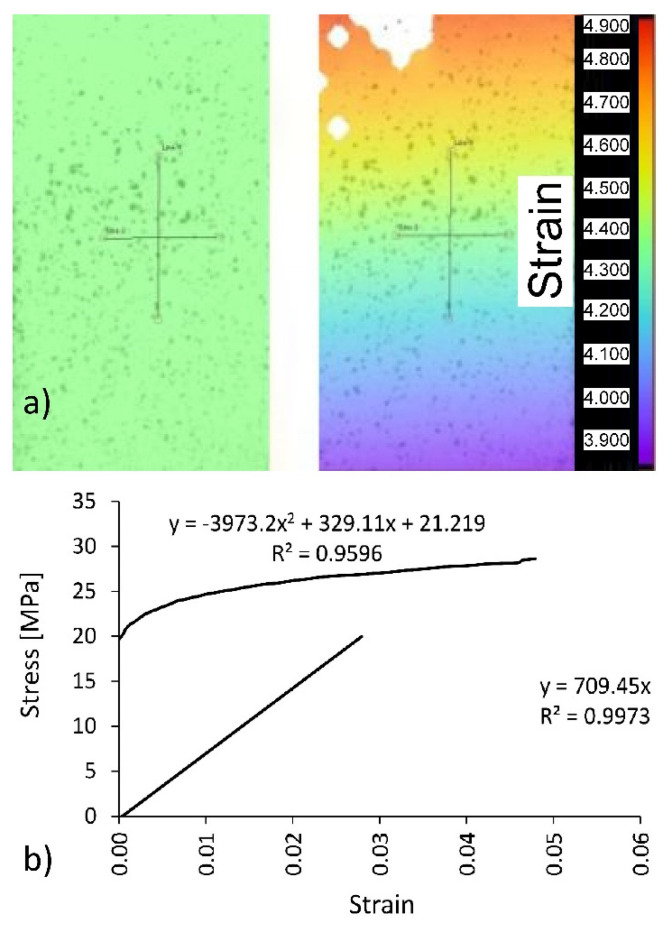
Measuring the displacements by using the pattern matching method: (**a**) virtual strain gauges, (**b**) limits of linear elasticity and plasticity for PA12.

Uniaxial tensile tests were performed on a 10 kN capacity numerically controlled Zwick 1445 universal testing machine (Zwick Roell AG, Ulm, Germany). The loading rate was 10 mm/min. In the tests, elongations and shortenings in the midpoint of the samples were recorded by Digital Image Correlation and Tracking method (DICT) using the Dantec system (Dantec Dynamics A/S, Skovlunde, Denmark) (Figure 2a).

In order to include plasticity in numerical calculations for selected dowels, the experimental stress–strain dependence had to be converted for polyamide (PA12) after the linear elastic range was exceeded (Figure 2b). First, the linear elastic range was determined to establish the linear equation for this section. As shown in Figure 2b, the slope of the straight line corresponds to the value of the modulus of linear elasticity for polyamide equal to *E* = 709 (standard deviation SD = 41) MPa, tensile strength *MOR* = 41 MPa (SD = 3.5 MPa), and Poisson’s ratio υ = 0.23 (SD = 0.01). Next, true stress $\sigma_{T}$ and the logarithmic plastic strain $\varepsilon_{L}$, required in the finite element method (FEM) algorithm, were calculated using the equations given below, in Equation (1)

(1) $$\varepsilon_{L}=\varepsilon_{T}-\left(\frac{\sigma_{T}}{E_{L}}\right)$$

where $\sigma_{T}=\sigma \left(1+\varepsilon \right)$, true stress, $\varepsilon_{T}=ln\left(1+\varepsilon \right)$ logarithmic strain, $E_{L}$ = modulus of elasticity of polyamide, σ = engineering stress, and ε = engineering strain. For the plastic range in Figure 2b above the straight line, the graph for $\sigma_{T}\;$= f($\varepsilon_{L}$) was plotted.

### 2.2. Design and Production of the Dowels

**Figure 3:**
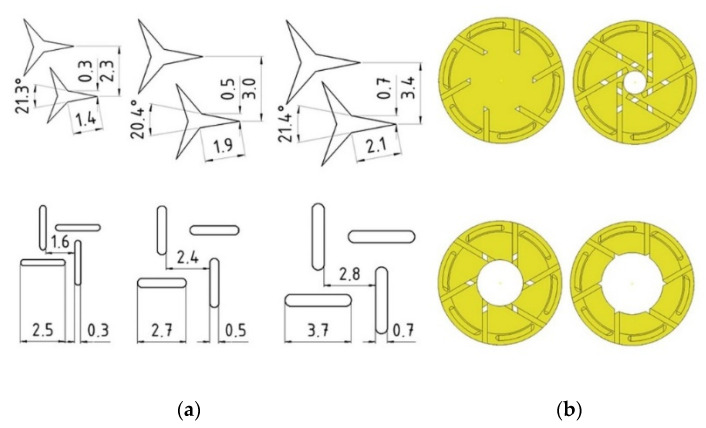
Dimensions (in mm) of auxetic inclusions (**a**) and cross-sections (**b**) of the dowels.

Engineered materials like lattice structures can be theoretically used to modify the local material properties and strength with minimization of the mass of components [30]. In work [31], a numerical approach was proposed to predict the mechanical response of lattice structures fabricated by means of a suitable additive layer manufacturing (ALM) process. Results show that both the relative density and the geometrical features of the representative volume element (RVE) strongly affect the equivalent macroscopic elastic behavior of the lattice. In particular, they are useful for modeling lightweight sandwich panels with a lattice core and facings [32,33,34,35,36,37,38,39,40,41,42,43]. However, the use of inclusions, as shown in Figure 3, should be considered for modeling dowels whose withdrawal strength is dependent on the mounting and friction forces [44,45,46,47,48].

In the study, it was planned to design 24 types of auxetic dowels with a different type (T: triangular, R: rectangular) and size (3: 0.3, 5: 0.5, 7: 0.7 mm) of inclusions, and different diameter of holes (A: 0, B: 1.5, C: 3, D: 4 mm), and a non-auxetic reference dowel (RF). Cross-sections and the inclusion sizes of designed dowels are given in Figure 3.

**Figure 4:**
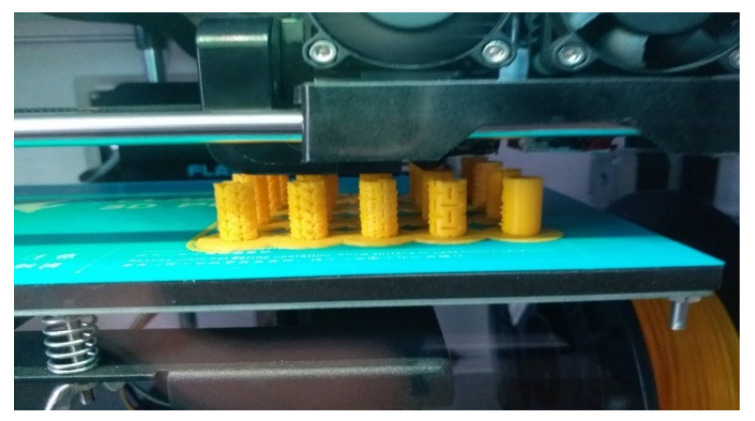
Pre-production of the dowels with 3D printing technology.

The inclusion size factor (3, 5, 7) was described as the dimension of a single gap of triangle or rectangle shape, respectively 0.3, 0.5, and 0.7 mm (Figure 3a). All the other dimensions are depending on these gaps and their periodicity. For the comparison, a non-auxetic dowel without inclusions and hole was designed as a reference. Prototype dowels were produced with 3D printing technology before the real dowels were produced (Figure 4).

The models were printed in the fused deposition modeling (FDM) technology with 3D Flashforge printers (FlashForge Corporation, Jinhua, Zhejiang, China). The used filament was polylactide (PLA) (3D Drukujesz, Poznan, Poland), and the printing temperature was 210 °C.

**Figure 5:**
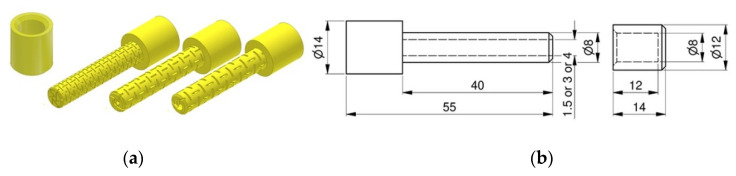
General view (**a**) and dimensions (**b**) of designed dowels and muff (in mm).

The importance of lightweight structures in many fields of engineering has been well known for a long time [49]. The innovations in technological processes based on material addiction allow pushing the design towards challenging geometries and associated structural properties. After the production of prototype dowels, the dowels with triangular inclusions have not been found to have sufficient strength and stiffness for furniture joints. The critical decision was made on the basis of an organoleptic evaluation. The dowels were easily bent in the hands under the influence of very small loads. At the same time, most of the dowels were breaking. Therefore, the study was continued using only 12 types of auxetic dowels with rectangular (rectangles with semicircles in two ends) inclusions. All manufactured dowels had a special head for easy application of the withdrawal force (Figure 5a).

Dowels were in 40 mm length and 8 mm diameter, while the muffs were in 14 mm length and 12 mm diameter (Figure 5b).

The selective laser sintering (SLS) printing technology and polyamide 12 (PA12) was utilized to produce the designed dowels. The default layer height used was 100–120 microns. The tensile specimen and the dowels were printed with the same parameters. The parameters were selected due to the producing capacity of the 3D printing machine Lisa Sinterit, (3D Center, Wrocław, Poland). In the production, firstly, 3D models of the designed dowels were modeled in the Autodesk Inventor software (Autodesk, Warszawa, Polska). Then, based on the CAD models, STP and STL files were prepared for numerical analyses and 3D printing, respectively. STP which is a file extension for a 3D graphic file used by CAD software and STL is a file format native to the stereo lithography CAD software created by 3D systems.

Totally, 130 dowels, including 13 different types of dowels and 10 replications for each, were produced. The diameter of dowels and the inner diameter of corresponding muffs were individually measured with a digital caliper. On this base, the average interference tolerance was calculated. These data were used in numerical analyses and theoretical calculations of contact pressure between the dowels and muffs. The final plan of the study and some measured average dimensions of the produced dowels and muffs are given in Table 1.

### 2.3. Mounting and Preparation of the Withdrawal Test Samples

**Figure 6:**
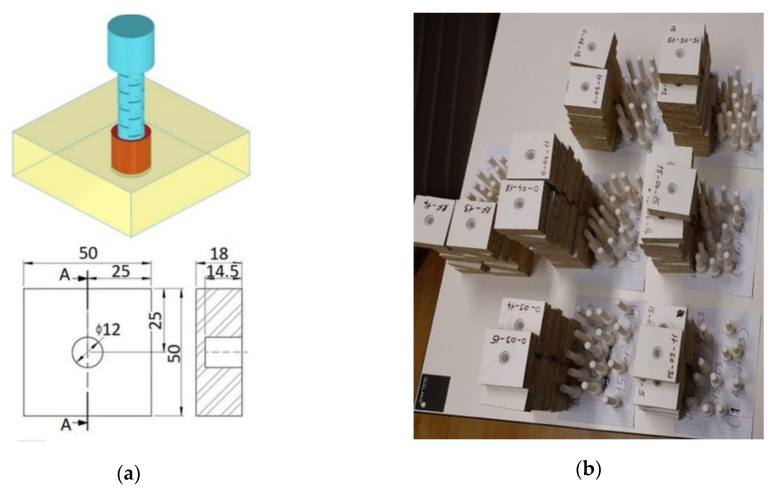
Dimensions (**a**) and real pictures (**b**) of all samples.

The sample substrate was made of particleboard 18 mm in thick, modulus of elasticity MOE = 2488 MPa (SD = 43 MPa), modulus of rupture MOR = 11.17 MPa (SD = 1.25 MPa, υ = 0.30). The muffs were glued to the particleboards by using Jowat^®^ UniPUR 687.22 adhesive (Jowat Swiss AG, Buchrain, Switzerland). Then, the dowel was fully inserted (10 mm) into the muff. The particleboard samples were 50 × 50 mm square and in 18 mm thick. The dimensions and real pictures of withdrawal test samples are shown in Figure 6.

Before the tests, withdrawal test samples were kept for at least two weeks in a conditioning chamber at 20 °C ± 2 °C and 65% ± 3% relative humidity.

**Figure 7:**
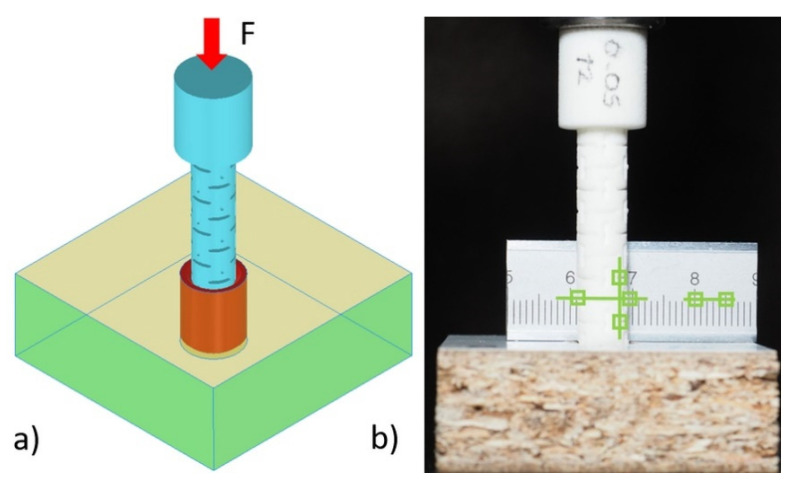
Geometry of samples for mounting tests: (**a**) loading, (**b**) measuring of strains.

Inserting of the dowels exactly 10 mm into the muffs were provided with the 10 kN capacity numerically controlled Zwick 1445 universal testing machine (Zwick Roell AG, Ulm, Germany) with a 10 mm/min loading rate under the static uniaxial loading before the withdrawal strength tests. In applying the mounting force to the dowels, the elastic properties (Poisson’s ratio) of dowels necessary for numerical modeling were determined with the digital image correlation (DIC) method. In the mounting, a reference ruler was placed behind the specimens. Before and during the loading, a few pictures of the samples were taken with an Olympus OM-D camera (Olympus, Tokyo, Japan). Next, dowel strains in vertical and horizontal directions were analyzed using the National Instruments IMAQ Vision Builder 6.1 software (National Instruments, Austin, TX, USA) (Figure 7).

Poisson’s ratios were calculated by applying the edge detection method in the digital image analysis.

### 2.4. Withdrawal Strength Testing

**Figure 8:**
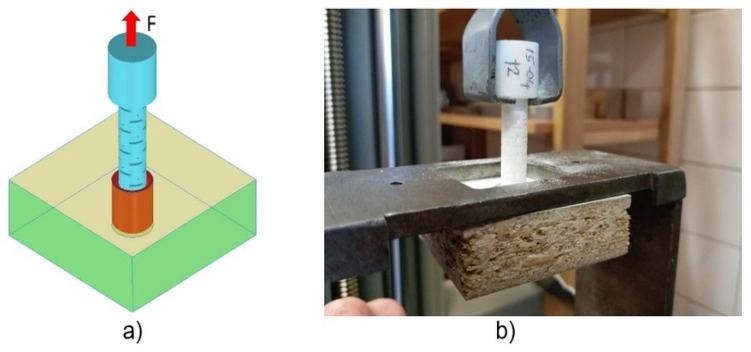
Geometry of samples for withdrawal tests: (**a**) loading, (**b**) real test.

Withdrawal strength tests of the dowels were also carried out on a 10 kN capacity numerically controlled Zwick 1445 universal testing machine (Zwick Roell AG, Ulm, Germany) with a 10 mm/min loading rate under the static uniaxial loading (Figure 8a).

Produced dowels had a special head for easy application of the load. In the tests, a concentrated static vertical tension load was applied from the dowel head as withdrawal force (Figure 8b). Withdrawal force was applied until the dowel exactly pull out from the muff. The withdrawal force (N) needed to pull out the dowel from the muff, and corresponding displacements were recorded with an accuracy of 0.01 N and 0.01 mm, respectively.

### 2.5. Mathematical Model of Dowel Withdrawal

**Figure 9:**
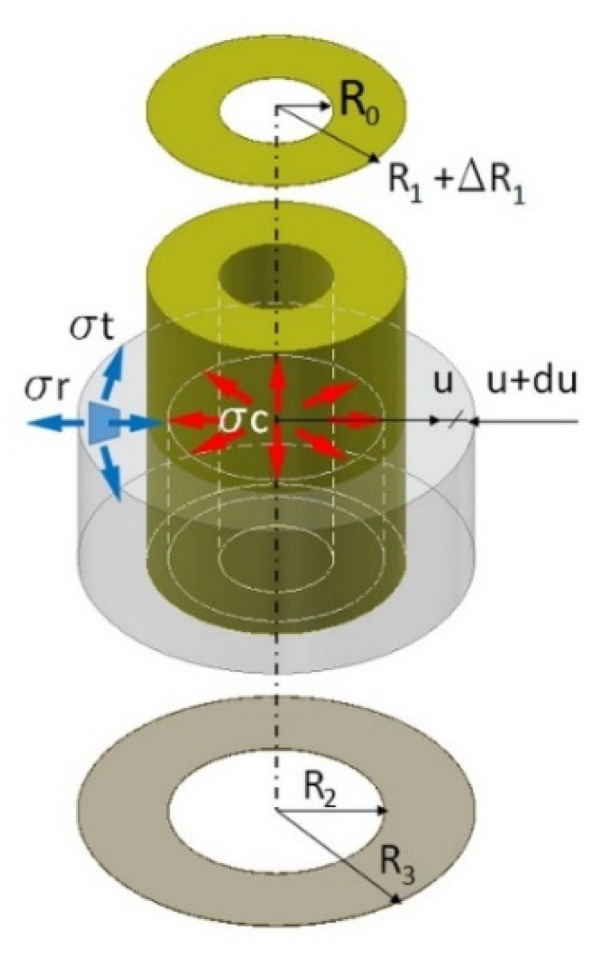
Model of the pressed joint with dowels have hole.

To determine the contact pressures in the pressed joint, (in this case also withdrawal force), the Lame analogy was used [30]. In the analyzed case, the muff has an outer radius $R_{3}$ (mm) and an inner radius $R_{2}$ (mm) (Figure 9).

The dowel has an outer radius $R_{1}$ + $∆R_{1}$ (mm), larger by $∆R_{1}$ (mm) then the inner radius of the muff $R_{2}$, and the radius of the central hole $R_{0}$ (mm).

The process of withdrawal of the dowel is equivalent to exerted contact stress (pressure) $\sigma_{c}$ (MPa) on the inner surface of the muff and the same stress on the outer surface of the dowel. The value of this stress was calculated from the assumption that $R_{1}=R_{2}$ due to the contact stress $\sigma_{c}$. Using the solution proposed by Lipka [50] circumferential stresses $\sigma_{t}$ (MPa) and radial stresses $\sigma_{r}$ (MPa) in the elementary segment of the muff can be expressed in the form, Equations (2) and (3)

(2) $$\sigma_{t}=\frac{E}{1-\vartheta^{2}}\left(\frac{u}{r}+\vartheta \frac{du}{dr}\right)$$

(3) $$\sigma_{r}=\frac{E}{1-\vartheta^{2}}\left(\frac{du}{dr}+\vartheta \frac{u}{r}\right)$$

where E (MPa)—module of elasticity, ϑ—Poisson’s ratio, du (mm)—an increment of displacement in the radius direction, u (mm)—displacement in the radius direction, dr (mm)—an increment of intermediate radius, and r (mm)—intermediate radius. For the connection as in Figure 7, the appropriate stresses can be presented by Lame equations [30], Equations (4) and (5)

(4) $$\sigma_{t}=\frac{\sigma_{c}R_{2}^{2}}{R_{3}^{2}-R_{2}^{2}}\left(1+\frac{R_{3}^{2}}{r^{2}}\right)-\frac{\sigma_{c}R_{3}^{2}}{R_{3}^{2}-R_{2}^{2}}\left(1+\frac{R_{2}^{2}}{r^{2}}\right)$$

(5) $$\sigma_{r}=\frac{\sigma_{c}R_{2}^{2}}{R_{3}^{2}-R_{2}^{2}}\left(1-\frac{R_{3}^{2}}{r^{2}}\right)-\frac{\sigma_{c}R_{3}^{2}}{R_{3}^{2}-R_{2}^{2}}\left(1-\frac{R_{2}^{2}}{r^{2}}\right)$$

hence the new outer radius of the dowel $R_{1}$ taking into account the displacement *u* for $r=R_{1}$ is equal to, Equation (6)

(6) $$R_{1}+∆R_{1}+u_{r=R_{1}}=R_{1}+∆R_{1}-\frac{\sigma_{c}R_{1}}{E}\left(\frac{R_{1}^{2}+R_{0}^{2}}{R_{1}^{2}-R_{0}^{2}}-\vartheta \right),$$

and inner radius of the muff $R_{2}$, taking into account the displacement *u* for $r=R_{1}$ is equal to, Equation (7)

(7) $$R_{2}=R_{1}+u_{r=R_{1}}=R_{1}+\frac{\sigma_{c}R_{1}}{E}\left(\frac{R_{3}^{2}+R_{1}^{2}}{R_{3}^{2}-R_{1}^{2}}+\vartheta \right)$$

Therefore, after comparing Equations (6) and (7), it can be obtained the value of contact pressures in the pressed joint can be derived as, Equation (8)

(8) $$\sigma_{c}=\frac{E∆R_{1}}{2R_{1}^{3}}\left(\frac{\left(R_{3}^{2}-R_{1}^{2}\right)\left(R_{1}^{2}-R_{0}^{2}\right)}{R_{3}^{2}-R_{0}^{2}}\right)$$

In the case of a joint in which the dowel has no hole, classic Hook’s law can be applied. Hence, using the markings as in Figure 9 and replacing the angular segment of the circle with a rectangular segment, contact pressures can be re-written in the form of, Equation (9)

(9) $$\sigma_{c}=\frac{∆R_{1}}{R_{1}+∆R_{1}}E.$$

Therefore, the withdrawal force F (N) can be calculated, taking into account the friction coefficient μ, Equation (10)

(10) $$F=2\mu \sigma_{c}\pi R_{1}L$$

where L (mm)—the length of the dowel in the muff.

In the planned task, the withdrawal force F was experimentally determined first. Then, the contact pressure value $\sigma_{c}$ was calculated numerically. On this basis, the coefficients of friction for individual types of connections were calculated from the equations as, Equation (11)

(11) $$\mu =\frac{F}{2\sigma_{c}\pi R_{1}L}$$

### 2.6. Numerical Model

**Figure 10:**
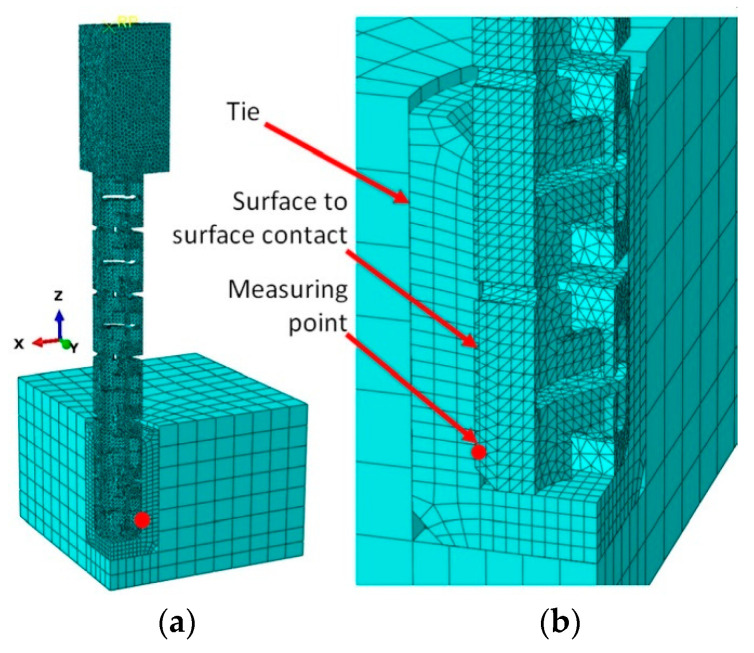
One-quarter of the FEM model (**a**) and contact surfaces in joints (**b**).

FEM models of withdrawal test samples were supported similarly as in the actual test. The geometry, loading, and boundary conditions of the model were based on Figure 6. In general, 10-node modified quadratic tetrahedron element C3D10M type, was used in all parts of model (about 700,000 elements and 1,600,000 nodes per model) (Figure 10).

While preparing the numerical model, the size and type of the finite element mesh were tested many times. For larger mesh sizes, the problem of creating little or zero-volume elements was encountered. They appeared mainly in a dowel, near the inclusions. This made the task unsolvable. Increasing the mesh density resulted in a long computation time exceeding 48 h on the computing cluster of the Supercomputing and Networking Center in Poznań. The tested density of the finite element mesh was considered final. Before analysis, the material properties of polyamide PA12 were calibrated. The standard functions of Abaqus were used for this purpose.

As shown in Figure 10b, the diameter of the dowel was larger than the inner diameter of the muff (Table 1). Therefore, during pulling out the dowel from the muff, firstly the contact pressure between outer surfaces of the dowel and inner surface of the muff were simulated, and then the withdrawal force increase caused by the interaction of contacting surfaces were calculated. The PA12 is modeled as elastic-perfectly plastic materials, while particleboard is modeled as elastic-isotropic material. In addition, geometric nonlinearity is considered to represent the large deformation of the structure. Between dowel and muff general surface contact were modeled, including no friction. The adoption of this hypothesis results from the described method of modeling the tight fit of a dowel and a hole. Due to this fit, the reaction of the deformed auxetic dowel will cause contact stresses between the surfaces of the dowel and the hole. During withdrawal, the dowel will slightly increase its diameter. Thus, the mutual contact pressure of these surfaces will determine the withdrawal strength of the dowel. Computations were performed at the Poznań Supercomputing and Networking Center (PSNC) using the Eagle computing cluster. The finite element analysis was conducted using Abaqus/Explicite v.6.14-2 (Dassault Systemes Simulia Corp., Waltham, MA, USA). The measuring point indicated in Figure 10b was used to determine the change in the value of the contact pressures during insertion of the dowel into the muff.

Based on the results of numerical calculations, contact pressures and coefficients of frictions of dowel joints were obtained and compared to real test results.

### 2.7. Evaluation of the Experimental Withdrawal Strength Results

Two different approaches were applied in the statistical evaluation of the withdrawal strength data. In the first approach, withdrawal strength values of the reference (non-auxetic) dowels were taken into account in the analyses; thus, the withdrawal strength differences of the 12 different auxetic dowels relative to the withdrawal strength of reference dowels were determined. For this approach, the one-way analysis of variances (ANOVA) general linear model procedure was performed to analyze the main effect (dowel type) on the mean of withdrawal strength. In the case of the second approach, the withdrawal strength values of the reference dowels were not taken into account in the analyses. This time, the effect of the two independent variables (dowel hole diameter and inclusion size) and their interaction (dowel hole diameter × inclusion size) on the withdrawal strength were investigated. For this approach, the two-factor analysis of variances (MANOVA) general linear model procedure was performed to analyze main factors and two-way interaction on withdrawal strength.

In both cases, the least significant difference (LSD) multiple comparisons procedure at a 5% significance level was performed to determine the mean differences of withdrawal strength values of the samples tested considering the ‘dowel type’ that was statistically significant in the ANOVA; and ‘dowel hole diameter’, ‘inclusion size’, and ‘dowel hole diameter’ × ‘inclusion size’ interaction that were statistically significant in the MANOVA results. Minitab (Version 17) statistical software was utilized for the statistical analyses (Minitab, LLC, State College, PA, USA).

## 3. Results and Discussion

### 3.1. Poisson’s Ratios of the Dowels

Poisson’s ratios of the designed dowels were numerically and experimentally determined. The Poisson’s ratios of each designed dowel are given in Table 2.

As seen in Table 2, the Poisson’s ratios calculated by numerical analyses and experimental results are consistent with each other. For the dowels without hole (A3, A5, A7), negative Poisson’s ratios were not obtained from both numerical and real tests. It means that these dowels did not show auxetic properties. From these results, it could be said that there should be a hole inside the dowels for providing the auxetic properties. In the case of the dowels with the hole (B3, B5, B7, C3, C5, C7, D3, D5, D7), the Poisson’s ratio values of each dowel were negative. This table also shows the consistency that with increasing inclusion sizes, Poisson’s ratios decrease. Also, in the case of dowels without holes, the auxetic structure of the outer surface of the dowel causes a decrease in Poisson’s ratio. Similarly, as the hole diameter in the dowel increases, the Poisson’s ratio decreases.

### 3.2. Experimental Results for Withdrawal Strength of Dowels

**Figure 11:**
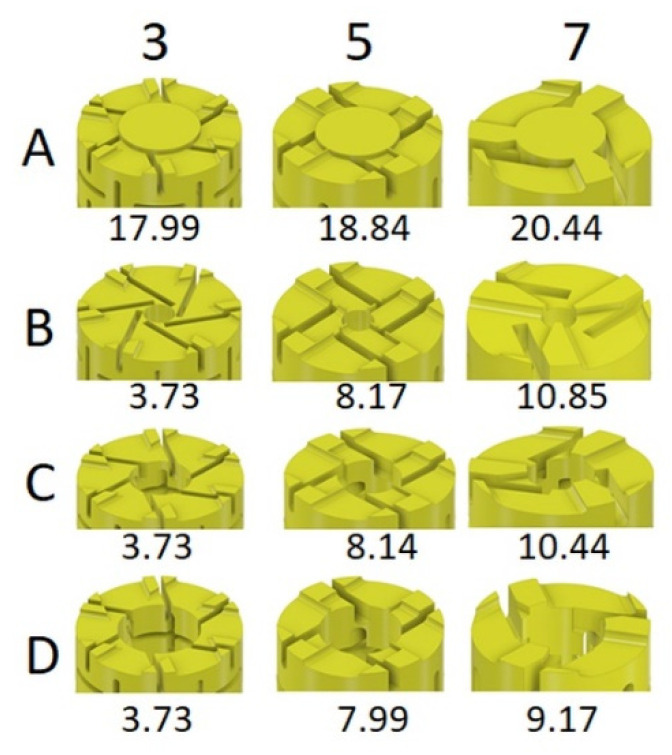
Cross-sectional area of dowels depends on the inclusion size and hole diameter (in mm^2^).

In the experiments, two different types of failure were observed in the form of the dowels pulled-out from the muff (RF, A3, A5, A7, B7, C7, D5, D7) or broken (B3, B5, C3, C5, D3). The experiments took 30–45 s if the dowels pulled out from the muff, while tests took just about 5–10 s if the dowels broke. It can be said that the failure type especially depends on the inclusion size. Generally, the dowels with small inclusions broke under the withdrawal force. This phenomenon can be explained by the fact that small inclusions decrease the cross-sectional area of these dowels (Figure 11).

The tension stresses occurred at the cross-sections of these dowels under the withdrawal force, exceeding the limit stress values of the PA12 material (MOR = 41 MPa). Therefore, these dowels broke before they were pulled out from the muff. In the case of dowels with a sufficient cross-section, they were pulled out from the muff.

It is also necessary to emphasize the second important factor resulting in the effect of destroying the dowels before pulling out from the muff. This factor is the shape of the cross-sectional area. The slender polygon-shaped surfaces (B3, C3, D3) favor stress concentration, which is an additional factor causing the dowel to break suddenly during pulling out. The cross-sectional surfaces of the pins B7, C7, and D7 are devoid of this defect, so the stresses do not tend to concentrate intensely.

The withdrawal strength results of one-way analysis of variance that was performed according to the first statistical approach are given in Table 3.

**Figure 12:**
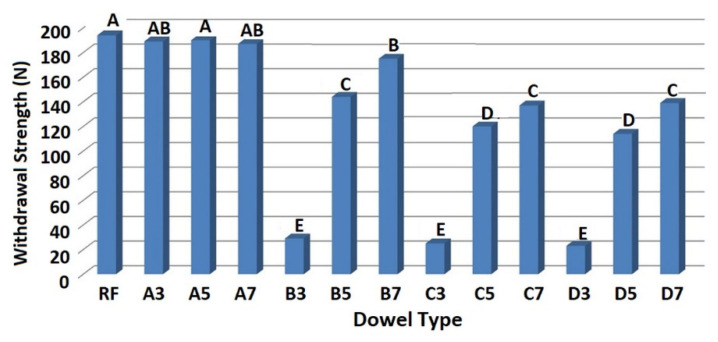
Mean comparison results of withdrawal strength values of the dowels.

According to the first statistical approach, the ANOVA results indicated that the main factor (dowel type) for mounting force values was statistically significant at the 5% significance level on withdrawal strengths of dowels. Table 4 gives the mean of withdrawal strength values of dowels with their coefficients of variation, and Figure 12 shows the LSD comparison homogeneity groups. Values followed by the same capital letter are not significantly different in Figure 12.

Generally, results indicated that the auxetic dowels gave lower withdrawal strength values than the non-auxetic dowels. Especially, the auxetic dowels with 0.3 mm inclusions (B3, C3, D3) gave very low withdrawal strength values (29 N, 25 N, 23 N) compared to the other groups. As seen in Table 4, COV values of the mentioned dowel groups are also too high. This result can be explained by the small cross-sectional areas of these auxetic dowels. Under the withdrawal force, the dowels exposed to the tensile stresses in the longitudinal direction and the cross-sectional areas of these auxetic dowels could not carry the occurred tension stresses. Moreover, increasing the dowel hole diameter has considerably reduced the cross-sectional area, and these dowels have become even weaker. The fact that the failure type observed in the experiments of these dowels was the breaking of dowels which confirmed this situation. The other important effective factors on withdrawal strength are the strength of the dowel material (PA12) and 3D printing technology of the dowels. According to the results, it can be concluded that the PA12 material with SLS printing technology is not suitable for producing the auxetic dowels. The modulus of elasticity of the materials produced with this technology decreases significantly. The dowels which have inclusions but without holes (A3, A5, A7) gave high withdrawal strength values (189 N, 190 N, 187 N) up to the RF (194 N) dowels. In the case of the auxetic dowels, only B7 gave withdrawal strength values (175 N) close to the non-auxetic dowels. According to results, there was no statistically significant difference between the auxetic B7 dowel and non-auxetic A7 and A3 dowels. The results of the two-factor analysis of variance for the second statistical approach are given in Table 5.

MANOVA results indicated that the main effects (dowel hole diameter and inclusion size) and their interaction (dowel hole diameter × inclusion size) for withdrawal strength values were statistically significant at the 5% significance level. Comparing the F-values to one another, it can be concluded that the withdrawal strength was mainly affected by the inclusion size. Table 6 gives mean comparisons of withdrawal force values of the dowels for dowel hole diameter and inclusion size.

Results indicated that the mean withdrawal strength values of dowels decreased as the dowel hole diameter is increased. The withdrawal strength of auxetic dowels with 1.5 mm hole (B) was lower than the withdrawal strength of non-auxetic dowels (A) by 38%. In case of the auxetic dowels with 3 mm hole (C) and 4 mm hole (D); the withdrawal strengths were considerably lower than the non-auxetic dowels (A), on average 50%. The withdrawal strength values between the dowels with 3 mm hole (C) and 4 mm hole (D) were not statistically different. In the case of inclusion size, 0.7 mm patterned dowels showed the greatest withdrawal strength values, while 0.3 mm patterned dowels gave the lowest values. The mean withdrawal strength values of 0.3 mm patterned dowels were considerably lower than the 0.5 mm and 0.7 mm patterned dowels by 52% and 57%, respectively. The withdrawal strength of 0.7 mm patterned dowels were higher than the withdrawal strength of 0.5 mm patterned dowels by only 11%. The two-way interaction of the withdrawal force of dowels according to the dowel hole diameter and inclusion size are given in Table 7.

As seen in Table 7; the non-auxetic dowels (A3, A5, A7) had the highest withdrawal strength values, whereas 0.3 mm patterned dowels with the hole (B3, C3, D3) had the lowest. Overall, it could be said from the results that; the dowels with holes that had auxetic properties gave lower withdrawal strength values than the dowels without holes that had non-auxetic properties. Only the B7 dowels gave withdrawal strength values close to the non-auxetic dowel groups. According to the results, the withdrawal strength differences between the B5, C7, and D7 dowels, and similarly between the C5 and D5 dowels were not statistically significant.

### 3.3. Results of the Numerical and Theoretical Calculations

**Figure 13:**
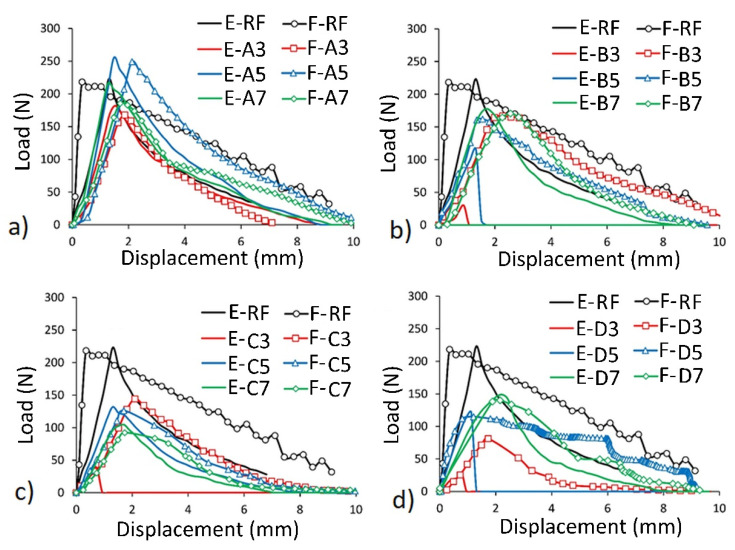
Relationship between load and displacement. Comparison of the experimental (**E**) and numerical (**F**) results: (**a**) dowels A type, (**b**) dowels B type, (**c**) dowels C type, (**d**) dowels D type.

The numerical and experimental load–displacement relationships of dowels under the withdrawal force compared to the reference dowel (RF) are given in Figure 13 for each group.

It should be noted that the presented comparison does not apply to mean values which were discussed in Section 3.2, but individual selected joints from each group. In this way, it was ensured that the numerical model corresponds to a specific dowel and a specific muff. Thus, the discussed withdrawal strength values differ from the mean values.

According to the load–displacement relationships; generally, the numerical (FEM) results gave reasonable estimates for the withdrawal strength values and mechanical behaviors of the dowels. All dowels showed similar behavior with the RF dowel, except for the B3, C3, and D3 dowels. The ultimate withdrawal strength values were achieved until approximately 2 mm displacement was reached. After this point, the withdrawal strength values of the dowels were getting lower. Especially, the FEM results of non-auxetic dowels without hole (A3, A5, A7) were consistent with the actual test results. In case of auxetic dowels, very close withdrawal strength values were obtained from the FEM, and actual test results for the 0.7 mm patterned dowels with 1.5 mm holes (B7), and 0.5 mm and 0.7 mm patterned dowels with 3 mm and 4 mm holes (C5, C7, D5, D7). For the 0.3 mm patterned auxetic dowels with any diameter of holes (B3, C3, D3), reasonable estimates were not obtained in terms of withdrawal strength and mechanical behavior from the FEM. As a matter of fact, the actual withdrawal strength values for these groups were much lower than that obtained from the numerical analyses.

It can be seen from the results that the non-auxetic dowels (A3, A5, A7) pulled out from the muff with higher withdrawal force values than the auxetic dowels (B3, B5, B7, C3, C5, C7, D3, D5, D7). The highest withdrawal strength values were obtained from the A5 dowels among all dowels.

**Figure 14:**
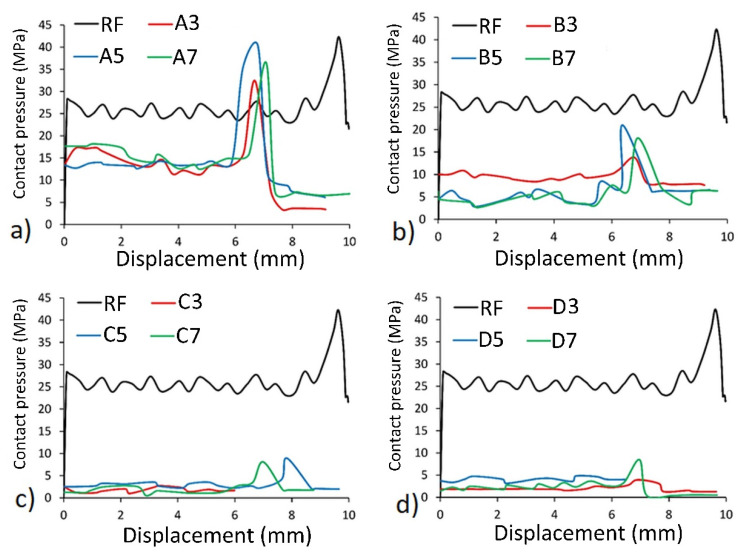
Relationship between contact pressure and displacement: (**a**) dowels A type, (**b**) dowels B type, (**c**) dowels C type, (**d**) dowels D type.

The contact pressures around the surface of dowels and displacement relations under the withdrawal force compared to the RF dowel were also investigated in the numerical analyses, and results are given in Figure 14 for each group.

A characteristic and repetitive feature of the curves are shown in Figure 14. As can be deduced, contact pressure-displacement relations were getting horizontal until approximately 6- and 9-mm displacement were provided for the dowels with inclusions (A, B, C, D) and RF dowels, respectively. After this point, the contact pressure values reached maximum values; and then, after about 1 mm displacement along with the maximum values, a sharp decrease in contact force was recorded. Generally, auxetic dowels (B, C, D), especially the dowels with 3 mm and 4 mm hole (C, D), withdraw from the muff under very low contact pressure values. The contact pressure values around the surface of the auxetic dowels were considerably lower than that of the RF dowel. Contact pressures around the surface of non-auxetic dowels (A3, A5, A7) were also lower than the RF dowel. However, the contact pressures were very close between the A5 and RF dowels. It can be said that higher contact pressure values caused higher withdrawal forces.

The diameter of the auxetic dowels expands under the withdrawal force because of their auxetic behavior. Although negative Poisson’s ratios were obtained from the dowels with inclusions and holes (B, C, D), the contact pressures around the outer surface of these dowels were not as high as expected.

**Figure 15:**
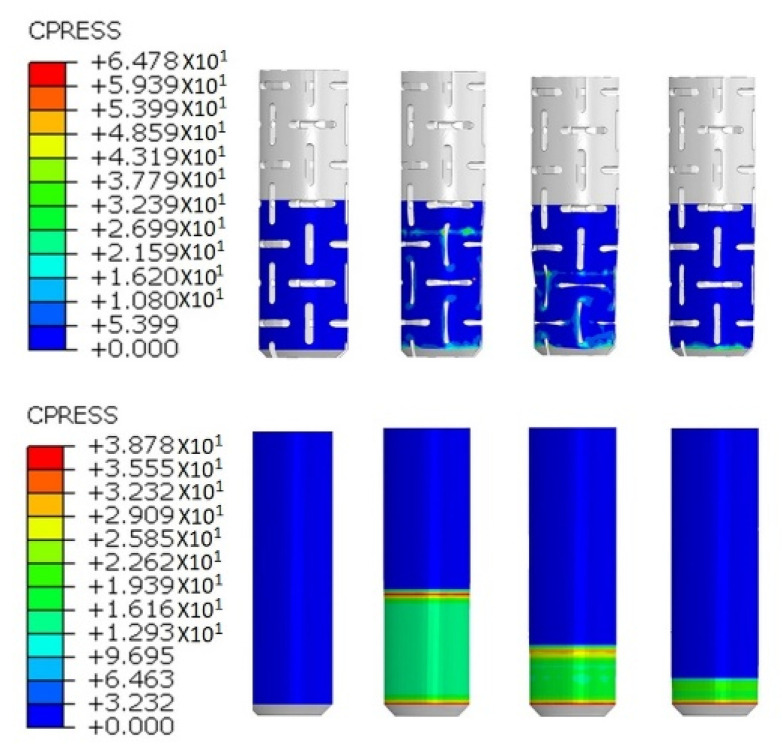
Contact pressure distribution during the withdrawal force: auxetic (**top**) and RF (**bottom**) dowels.

The contact pressure distribution around the surface of auxetic and RF dowels during the withdrawal force was also observed from the numerical analyses, and the graphical results are given step by step in Figure 15.

According to the numerical analyses results, contact pressure around the surface of the auxetic and RF dowels remained at the same level as the dowel withdrawal from the muff approximately by 6 mm and 9 mm, respectively. The contact pressures around the surface of RF dowels were considerably higher than the auxetic dowels. Figure 15 also shows that the highest contact pressure occurs at the edges of the muff and the dowel; i.e., at the beginning and end of the contact surface. In addition, in the case of the reference dowel (RF) the distribution is even; whereas, on the surface of the auxetic dowel, it is concentrated near the inclusions.

**Figure 16:**
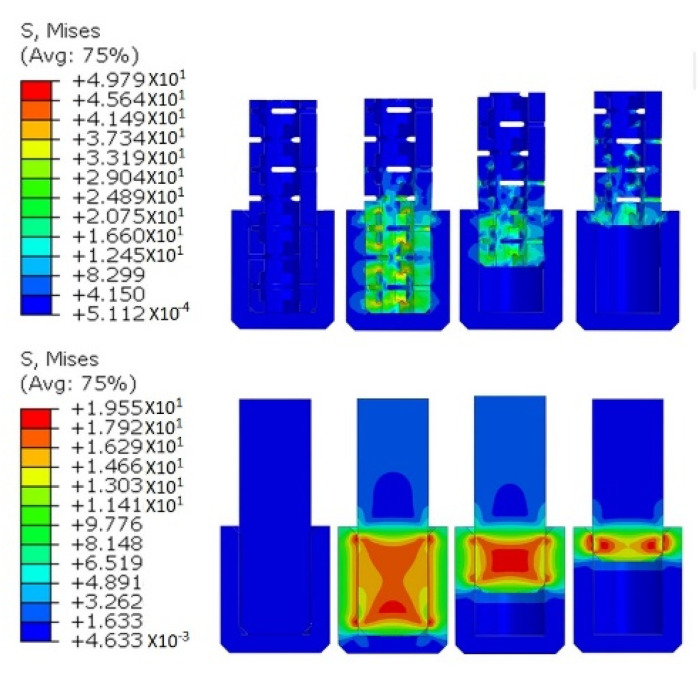
Stress distribution of dowels during the withdrawal forces for auxetic (**top**) and RF (**bottom**).

The von Mises stress distribution inside the auxetic and RF dowels during the withdrawal force were obtained from the numerical analyses, and the graphical results are given step by step in Figure 16.

As expected, stress distribution inside the dowels gave similar results to the distribution of contact pressure around the surface of dowels. As seen in Figure 16, the stress values inside the RF dowels were higher than the auxetic dowels. With the effect of withdrawal force, the stresses inside the surface of the RF dowels almost uniformly distributed along the central part of the dowel and remained at the same values as the dowel completely withdrew from the muff. In the case of the auxetic dowels, the stresses concentrated near the inclusions and gradually decreased during the withdrawal. Generally, the occurred stresses inside the auxetic dowels, especially the dowels with 0.3 mm inclusions were greater than the maximum strength of the materials because of the small cross-sections; therefore, the auxetic dowels broke during the withdrawal tests. For the RF dowels, the stresses were not greater than the maximum strength of the materials; thus resulting in the complete pull out of the RF dowels from the muff without breaking in the tests.

According to the results obtained from contact pressures and stress distributions, it could be concluded that more withdrawal force values were required to withdraw the non-auxetic dowels from the muff than auxetic dowels.

Theoretical calculations were performed by using the Lame analogy (Equation (8)) for the dowel with the hole, and by using the classic Hook’s law (Equation (9)) for the dowel without the hole. The withdrawal strength, contact pressure values, and friction coefficients obtained from the numerical analyses and the values experimentally obtained and theoretically calculated are presented in Table 8 for one sample according to each group.

Experimental and numerical results of withdrawal strength values are very close except for the dowels with 0.3 mm inclusions (B3, C3, D3). In the tests, the mentioned dowels broke because of their small cross-sectional area. Therefore, it can be said that the obtained experimental values do not reflect the withdrawal strength of the dowels. In conclusion, the FEM results gave reasonable estimates for the withdrawal strength values. In the case of the contact pressures and friction coefficients, it can be said that the numerical analysis and theoretical calculation results are consistent with each other as well. Therefore, considering the good compliance of the results of numerical calculations with the experiment, for further research, it was decided to use the results of numerical calculations.

## 4. Conclusions

This study was conducted to experimentally and numerically analyze the withdrawal strength of different kinds of auxetic dowels produced from PA12. In the scope of the study, it was planned to design 24 types of auxetic dowels with different types and sizes of inclusions, and different diameters of holes, and a non-auxetic reference dowel (RF). However, after the production of prototype dowels, dowels with triangular inclusions have been found to have insufficient strength and stiffness for furniture joints. Therefore, the study was continued using only 12 types of auxetic dowels with rectangular inclusions.

According to the numerical and experimental results, it should be noted that there should be a hole inside the dowels for obtaining a negative Poisson’s ratio. As the hole diameter and sizes of inclusions increases, the Poisson’s ratio decreases from −0.153 to −0.442. Also, dowels with small inclusions broke under the withdrawal force because small inclusions decrease the cross-sectional area of these dowels and favor stress concentration. In the tests, auxetic dowels gave considerably lower withdrawal strength values than the non-auxetic dowels. The inclusion size and diameter of the hole of auxetic dowels significantly affected the withdrawal strength values at the 5% significance level. Withdrawal strength of dowels decreased as the inclusion size is decreased or the dowel hole diameter is increased. The auxetic B7 dowels had the highest withdrawal strength values (175 N), whereas the auxetic B3, D3, and C3 dowels had the lowest (29 N, 23 N, 25 N, respectively).

Contact pressure between dowels and holes in the reference dowels were considerably higher (from two to six times) than that of auxetic dowels. In this case, it means that the withdrawal force was the highest, ranging from 190 N to 194 N. Analogical the von Mises stresses inside of the reference dowels were considerably higher than that of auxetic dowels. Therefore, lower withdrawal strength values were required to pull out the auxetic dowels from the muff. The results showed that numerical and theoretical analyses of the samples by FEM provide reasonable estimates of the actual test results.

The results of this study provide numerical and experimental information on the withdrawal strength of auxetic dowels. The auxetic dowels tested in this study did not show sufficient withdrawal strength values as expected. Therefore, it could be suggested that for future studies, the auxetic dowels should be produced with stronger materials and/or alternative 3D printing technologies should be experimented for production of the auxetic dowels. By improving SLS printing technology, the printed elasticity modulus can be enhanced so as to close to ‘real’ elasticity modulus. According to the results of [51], elastic properties of elements obtained via SLS 3D printing technology can be improved by using higher energy density, considering only the laser beam power variation.

## Figures and Tables

**Table 1:** Plan of the study and some measured dimensions of produced dowels and muffs.

Dowel Type	Hole Type	Inclusion Size	Mean Diameter of Dowels (mm)	Mean inner Diameter of Muffs (mm)	Average Interference Tolerance (mm)	Dowel Code
Non-auxetic	RF	Plain	7.86	7.59	+0.27	RF
Auxetic	A	3	7.88	7.50	+0.38	A3
		5	7.91	7.56	+0.35	A5
		7	7.90	7.56	+0.34	A7
	B	3	7.89	7.52	+0.37	B3
		5	7.89	7.54	+0.35	B5
		7	7.89	7.49	+0.39	B7
	C	3	7.87	7.56	+0.31	C3
		5	7.87	7.58	+0.29	C5
		7	7.86	7.52	+0.35	C7
	D	3	7.84	7.54	+0.30	D3
		5	7.86	7.59	+0.27	D5
		7	7.87	7.55	+0.32	D7

**Table 2:** Poisson’s ratio values of the dowels from numerical analyses and experiments.

Dowel Type	Dowel Code	Numerical Analyses (ϑ_n_)	Experiment (ϑ_t_)	Difference (%)
Non-auxetic	RF	0.219	0.230	−4.8
	A3	0.217	0.227	−4.4
	A5	0.207	0.214	−3.3
	A7	0.146	0.133	9.8
Auxetic	B3	−0.147	−0.153	−3.9
	B5	−0.213	−0.201	6.0
	B7	−0.234	−0.256	−8.6
	C3	−0.185	−0.182	1.6
	C5	−0.236	−0.229	3.1
	C7	−0.311	−0.346	−10.1
	D3	−0.336	−0.357	−5.9
	D5	−0.377	−0.388	−2.8
	D7	−0.412	−0.442	−6.8

**Table 3:** Summary of ANOVA results for withdrawal strengths according to the first approach.

Source	Degrees of Freedom	Sum of Squares	Mean Squares	F-Value	*p*-Value
Dowel type	12	496,187	41,348.9	171.18	0.000
Error	117	28,261	241.5		
Total	129	524,448			

**Table 4:** Mean withdrawal strengths of the dowels with their coefficients of variation.

Dowel Type	RF	A3	A5	A7	B3	B5	B7	C3	C5	C7	D3	D5	D7
Mean (N)	194	189	190	187	29	144	175	25	120	137	23	114	139
COV * (%)	10.4	6.5	8.6	17.9	31.5	8.7	8.5	32.2	6.9	11.9	23.5	8.3	14.9

*: Coefficients of variation.

**Table 5:** Summary of MANOVA results for withdrawal strengths according to the second approach.

Source	Degrees of Freedom	Sum of Squares	Mean Squares	F-Value	*p*-Value
Dowel hole diameter	3	184,307	61,435.7	269.33	0.000
Inclusion size	2	194,471	97,235.3	426.28	0.000
Hole diameter × Inclusion size	6	70,549	11,758.2	51.55	0.000
Error	108	24,635	228.1		
Total	119	473,962			

**Table 6:** Mean comparisons for dowel hole diameter and inclusion size on withdrawal strength.

Dowel Hole Diameter	Withdrawal Strength (*N*)		Inclusion Size	Withdrawal Strength (*N*)	
	Mean	(HG)		Mean	(HG)
(A) 0 mm	188	A			
(B) 1.5 mm	116	B	(3) 0.3 mm	67	C
(C) 3 mm	94	C	(5) 0.5 mm	142	B
(D) 4 mm	92	C	(7) 0.7 mm	159	A

**Table 7:** Comparison test results for dowel hole diameter–inclusion size interaction.

Dowel Hole Diameter	Inclusion Size					
	(3) 0.3 mm		(5) 0.5 mm		(7) 0.7 mm	
	Mean	(HG)	Mean	(HG)	Mean	(HG)
(A) 0 mm	189	A	190	A	187	AB
(B) 1.5 mm	29	E	144	C	175	B
(C) 3 mm	25	E	120	D	137	C
(D) 4 mm	23	E	114	D	139	C

**Table 8:** Withdrawal strength, contact pressures, and friction coefficients of the dowels.

Dowel Type	Withdrawal Strength (N)		Contact Pressures (MPa)		Friction Coefficients	
	FEM	Experimental	FEM	Theoretical	FEM	Theoretical
RF	218	223	20.8	22.5	0.037	0.033
A3	168	182	17.1	34.2	0.049	0.025
A5	249	256	13.3	31.4	0.121	0.052
A7	185	217	17.6	30.5	0.065	0.037
B3	167	30	10.0	1.7	0.066	0.384
B5	162	116	2.8	1.6	0.217	0.657
B7	171	178	4.4	1.8	0.189	0.490
C3	144	38	1.8	1.3	0.139	0.193
C5	126	132	2.6	1.1	0.145	0.367
C7	92	104	1.3	1.4	0.292	0.244
D3	81	27	1.9	1.0	0.205	0.335
D5	115	122	3.7	0.9	0.102	0.458
D7	140	149	1.8	1.0	0.227	0.390
